# Single-cell qPCR facilitates the optimization of hematopoietic differentiation in hPSCs/OP9 coculture system

**DOI:** 10.1590/1414-431X20187183

**Published:** 2018-03-15

**Authors:** Haide Chen, Mengmeng Jiang, Lei Xiao, He Huang

**Affiliations:** 1 College of Animal Science, Zhejiang University, Hangzhou, China; 2 School of Medicine, Zhejiang University, Hangzhou, China; 3 The 1st Affiliated Hospital, Zhejiang University School of Medicine, Hangzhou, China

**Keywords:** Single-cell qPCR, Human pluripotent stem cells, Hematopoietic differentiation, OP9-GFP, Transcription factors

## Abstract

Human pluripotent stem cells (hPSCs)/OP9 coculture system is a widely used hematopoietic differentiation approach. The limited understanding of this process leads to its low efficiency. Thus, we used single-cell qPCR to reveal the gene expression profiles of individual CD34^+^ cells from different stages of differentiation. According to the dynamic gene expression of hematopoietic transcription factors, we overexpressed specific hematopoietic transcription factors (Gata2, Lmo2, Etv2, ERG, and SCL) at an early stage of hematopoietic differentiation. After overexpression, we generated more CD34^+^ cells with normal expression level of *CD43* and *CD31*, which are used to define various hematopoietic progenitors. Furthermore, these CD34^+^ cells possessed normal differentiation potency in colony-forming unit assays and normal gene expression profiles. In this study, we demonstrated that single-cell qPCR can provide guidance for optimization of hematopoietic differentiation and transient overexpression of selected hematopoietic transcription factors can enhance hematopoietic differentiation.

## Introduction

Since the establishment of human pluripotent stem cells (hPSCs) in 1998 (1–3), it is well known that hPSCs can differentiate into a variety of tissue cells for replacement therapy of human diseases and for studying development. Many differentiation strategies have been established for various tissue cells, such as lung organoids (4), corneal epithelial cells (5), intestinal tissue (6), retinal tissue (7), liver (8), and blood cells (9). The transplantation of blood cells offers hope for the treatment of a variety of blood and immune system diseases. Because of the critical shortage of blood cells in clinical settings, several strategies have been established to generate hematopoietic stem cells (HSCs) and mature blood cells from hPSCs (10,11).

The hPSCs/OP9 coculture system is a widely used approach to generate CD34^+^ hematopoietic cells from hPSCs without additional cytokine (9,12,13). Our limited understanding of this differentiation process is responsible for the low efficiency of CD34^+^ hematopoietic cell production (only 10–15% are CD34^+^). Thus, it is necessary to analyze the differentiation process in detail for further optimization. Signaling molecules provided by OP9 have been identified and analyzed by proteomic analysis (14). Molecular profiling reveals gene expression characteristics of various hematopoietic progenitors identified by hematopoietic markers (15,16). However, these studies provide limited guidance for further optimization.

Recent advances in single-cell-based gene expression analysis allow us to reveal the dynamic gene expression of individual *CD34* ^+^ cells derived from hPSCs (17). Single-cell qPCR, which can analyze specific gene expression at the single-cell level, has been used to reveal the heterogeneity of blood cells effectively (18,19). In our study, single-cell qPCR was used to track the dynamic gene expression of individual *CD34* ^+^ cells from different stages of differentiation. We clustered *CD34* ^+^ cells according to the different gene expression characteristics. After transient overexpression of hematopoietic transcription factors (TFs), we generated more *CD34* ^+^ cells with normal expression level of *CD43* and *CD31*, which were used to define hematopoietic progenitors in hPSCs/OP9 coculture system (15). Furthermore, these *CD34* ^+^ cells possessed normal differentiation potency in colony-forming unit (CFU) assays and normal gene expression profiles in single-cell gene expression analysis.

The purpose of our study was to optimize hematopoietic differentiation in hPSCs/OP9 coculture system.

## Material and Methods

### Cell culture

hPSCs (NIH Codes: WA01, H1) were maintained on irradiated CF1 feeder cells as described in our previous paper (20). Before lentiviral transduction and hematopoietic differentiation, hPSCs were maintained in chemically defined mTeSR™1 medium (StemCell Technologies, Canada) without feeder as described previously (21). The 293T cells were maintained in DMEM medium (Invitrogen, USA) supplemented with 10% fetal bovine serum (FBS, Hyclone, USA). GFP-labeled OP9 (OP9-GFP) cells were maintained in α-MEM medium (Gibco, USA) supplemented with 20% FBS (Gibco) (12). hPSCs (H1 or H1+TFs) monolayers at 70% confluence were cultured in X-Vivo 15 medium (Lonza, Switzerland) supplemented with 1 μg/mL doxycycline (Dox), 1 mM sodium pyruvate (Sigma-Aldrich, USA), 1x non-essential amino acids (Invitrogen), 2 mM L-glutamine (Invitrogen), 50 mM 2-mercaptoethanol (Sigma-Aldrich), and the four growth factors, including 50 ng/mL recombinant human bone morphogenetic protein-4 (rhBMP-4, R&D, USA), 50 ng/mL recombinant human vascular endothelial growth factor (rhVEGF, R&D), 50 ng/mL recombinant human granulocyte-macrophage colony-stimulating factor (rhGM-CSF, R&D), and 20 ng/mL recombinant human stem cell factor (rhSCF, R&D). After a 4-day culture, cells were collected for flow cytometry analysis.

### Gene cloning and lentiviral vectors construction

The gene sequences were downloaded and managed by CLC sequence Viewer 7 (Qiagen, Germany) for primers design. Pfx (Invitrogen) was used to amplify gene open reading frames (ORFs) from 10-day Embryoid Body cDNAs of H1 or cDNAs bought from YouBio (China). ORFs were cloned into pEASY-Blunt Simple Vectors (Transgen Biotech, China) for sequence verification. Correct ORFs were subcloned into our Lv-ef1α-eGFP-tre-genes backbone (22). The cloned genes are listed in Supplementary Table S1.

### Lentiviral packaging

We packaged lentivirus as previously described (22). Lentiviral vectors and two helper vectors (Δ8.91 and pVSVG) were transfected into 293T with FuGENE HD (Roche, Switzerland). Packaged lentiviral units were collected at 48 and 72 h post-transfection. After titering, lentiviral units were stored in -80°C.

### Lentiviral transduction of hPSCs

After rinsing with PBS, hPSCs of 70% confluence were disaggregated to single cells by Accutase (Gibco). Cells (4×10^5^) were seeded on Matrigel (BD, USA) coated 6-well plates with 2 mL of mTeSR™1 medium supplemented with 10 μM Y-27632 ROCK inhibitor (Selleck, USA). After 48-h incubation, the original medium was replaced with fresh virus-containing mTeSR™1 medium (multiplicity of infection of 5–10) supplemented with 10 μg/mL polybrene (Sidansai, China). Virus-containing medium was removed 12 h after transduction. Lentivirus of specific genes (Lv-ef1α-eGFP-tre-genes) and rtTA (Lv-ef1α-rtTA-IRES-puro) were transduced simultaneously and 0.5 μg/mL puromycin was used to enrich positive cells. Genomic PCR was used to confirm virus integration. Forty-eight hours after treatment with 1 μg/mL Dox, qPCRs were used to confirm the transgene mRNA expression. Primers are listed in Supplementary Tables S2 and S3.

### Reverse transcription-polymerase chain reaction (RT-PCR) and real-time polymerase chain reaction (qPCR)

Total RNA prepared by RNeasy kit (Qiagen) was used as the templates for RT-PCR as described in our previous paper (23). qPCR was performed in LightCycler 480 (Roche) with SYBR Green-based PCR Master mix (Toyobo, Japan). Ct (threshold cycle) values of samples were analyzed by ΔΔCt method with *ACTB* as reference gene. The primers are listed in Supplementary Tables S3 and S4.

### Hematopoietic differentiation of hPSCs in hPSCs/OP9 coculture system

We induced hematopoietic differentiation in hPSCs/OP9 coculture system as previously reported (9). After 30 min Dispase (Invitrogen) treatment, attached hPSCs colonies were curled-up and collected into 15-mL centrifuge tubes. Colonies were dissociated into small cell clumps with gentle pipetting. After washing 3 times, cell clumps were resuspended in differentiation medium (α-MEM supplemented with 10% FBS and 100 μM MTG; Sigma-Aldrich). Overgrown OP9-GFP was prepared in 6-well plates before differentiation. The original medium was replaced with 2 mL differentiation medium before hPSCs seeding. hPSCs (2×10^5^) were seeded on each well of overgrown OP9-GFP covered 6-well plates. The next day (day 1), the original medium was replaced with 4 mL of fresh differentiation medium. At days 4 and 6, half of the medium was replaced with fresh medium. At days 8–9, the medium was collected into 15-mL centrifuge tubes and 2 mL 1 mg/mL Collagenase IV (Gibco) was added per well of 6-well plates and incubated for 30 min to digest the collagen-rich matrix. Collagenase IV was collected into 15-mL centrifuge tubes used previously. One milliliter 0.25% Trypsin/EDTA (Gibco) was added per well. After 15–20 min of incubation, 2 mL Recommended Medium (StemCell Technologies) was added to stop digesting. After pipetting, single cells were collected into 15-mL centrifuge tubes used previously. Cells were washed and resuspended with Recommended Medium for flow cytometry analysis. *CD34* ^+^ cells were enriched using EasySep™ *CD34* positive selection kit (StemCell Technologies) for CFU assays, single-cell qPCR, and flow cytometry analysis.

### Flow cytometry analysis of cell phenotype

Cells suspended in Recommended Medium were labeled with antibodies at 4°C for 30 min. Antibodies used were PE-Cy™7 Mouse Anti-Human *CD34* (BD), PE anti-human *CD43* (BioLegend, USA), and PE anti-human *CD31* (BioLegend). After staining, cells were analyzed by Cytomics™FC 500 (Beckman, USA) with FlowJo software (Tree Star, USA).

### Single-cell specific target amplification

Primers pool was prepared as described previously (18). Primers used are listed in Supplementary Table S4. Individual cells were picked up into 8-strip PCR tubes with 5 µL RT-PreAmp Master Mix (1.9 µL nuclease free water, 2.5 µL Reaction Mix, and 0.1 µL RT/Taq enzyme were mixed with 0.5 µL primers pool; Single Cell Sequence Specific Amplification Kit, Vazyme, China) by special Pasteur pipettes (Brand, Germany). Eight-strip PCR tubes were immediately frozen in -80°C refrigerator for 2 min. After brief centrifugation (300 *g*, 4°C, 3 min), tubes were immediately moved to PCR machine following kit instructions. After preamplification, samples were diluted 100-fold with double distilled water prior to qPCR.

### Gene expression analysis using single-cell qPCR

Single-cell qPCR was performed in LightCycler 480 (Roche) with SYBR Green-based PCR Master Mix (Toyobo). The primers are listed in Supplementary Table S4. A background Ct (30) was used for single-cell samples corresponding to log2 gene expression (log2 gene expression=30-Ct). Single-cell samples with outliers of *ACTB* gene expression were removed from the dataset. MeV (MultiExperiment Viewer, Dana-Farber Cancer Institute, USA) was used for analysis of hierarchical clustering (HCL) and non-negative matrix factorization (NMF). The ggplot2 and base plot package of R software (R Core Team, New Zealand) were used for plot drawing.

### CFU assays

CFU assays were performed using MethoCult™ H4435 Enriched (StemCell Technologies) following manufacturer's instructions. Three milliliters MethoCult™ with 5×10^3^/mL *CD34* ^+^ cells and penicillin-streptomycin were added onto 35-mm low-adherent plastic dishes. CFUs were counted and identified after 10–14 days of incubation according to the guide provided by StemCell Technologies.

### Statistical analysis

Data were analyzed by Prism 5 software (GraphPad, USA). Data are reported as means±SE. Significant differences were based on P<0.05 for all experiments.

## Results and Discussion

### Hematopoietic differentiation of H1 in hPSCs/OP9 coculture system

**Figure 1.**
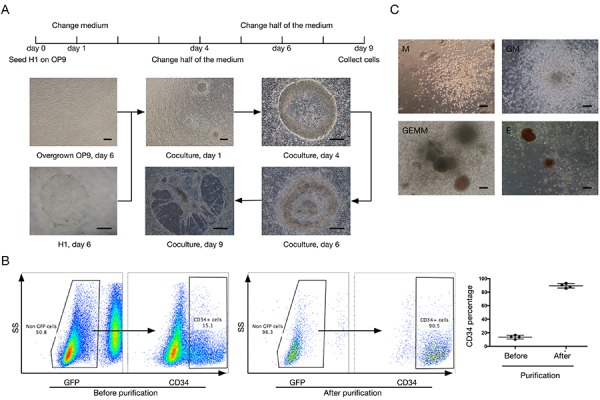
Hematopoietic differentiation of H1 cells in human pluripotent stem cells (hPSCs)/OP9 coculture system. *A,* Process flow diagram of hematopoietic differentiation in hPSCs/OP9 coculture system. Day 6 H1 were seeded on day 6 OP9. Morphological change of H1 clones is shown below. Scale bar=300 μm. *B,* The differentiated cells collected at day 9 were analyzed by flow cytometry. *CD34* ^+^ cells were highly enriched by magnetic cell sorting (from 13.53±1.34 to 89.25±1.60%). *C,* Morphology of different colony-forming unit types, including M, GM, GEMM, and E. Scale bar=100 μm.

Hematopoietic differentiation ability of H1 in hPSCs/OP9 coculture system was confirmed (Figure 1A) as described previously (12). Using flow cytometry analysis, we could easily distinguish H1 from OP9 because of the expression of GFP in OP9. After 8–9 days of differentiation, there were 10–15% *CD34* ^+^ cells. After magnetic cell sorting, the isolated *CD34* ^+^ cells, which ranged around 90% of the enriched fraction, were used in CFU assays and the following experiments (Figure 1B). The morphology of different CFUs was observed, including macrophage (M), granulocyte and macrophage (GM), granulocyte, erythroid, macrophage, and megakaryocyte (GEMM), and erythroid (E) (Figure 1C). Thus, our H1/OP9 coculture system was stable for the following study of hematopoietic differentiation.

### Single-cell gene expression analysis of CD34+ cells derived from H1/OP9 coculture system

**Figure 2.**
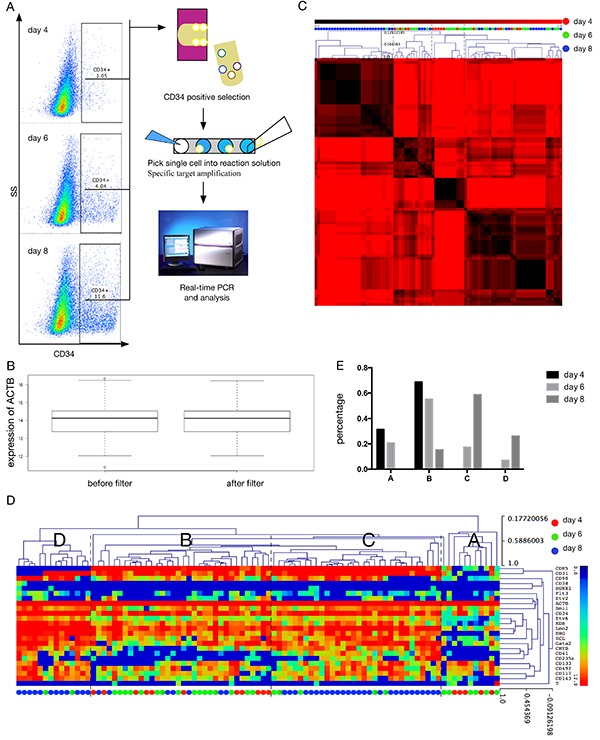
Single-cell gene expression analysis of *CD34* ^+^ cells derived from H1/OP9 coculture system. *A,* Process flow diagram of single-cell gene expression analysis. Individual *CD34* ^+^ cells were collected at days 4, 6, and 8. *B,* Samples were filtered based on the expression level of *ACTB* (log2 gene expression=30-Ct); outliers were removed. *C,* Heatmap of NMF showing cell-to-cell correlation. Red, green, and blue circles of each column correspond to individual *CD34* ^+^ cells from days 4 (n=16), 6 (n=29), and 8 (n=46), respectively; rank is 4. *D,* Heatmap of hierarchical clustering showing 4 clusters (A, B, C, and D) of *CD34+* cells. Red, green, and blue circles of each column correspond to individual *CD34* ^+^ cells from days 4, 6, and 8, respectively. Each row corresponds to a specific gene. Color scale was set from 0 to 12. Blue to green suggests low to moderate gene expression (log2) and green to red suggests moderate to high gene expression (log2). *E*, Distribution of samples (days 4, 6, and 8) in cluster A, B, C, and D.

To study the process of hematopoietic differentiation in H1/OP9 coculture system, we used single-cell gene expression analysis. *CD34* ^+^ cells appeared as early as days 3 and 4 of coculture and the proportion increased during differentiation (Figure 2A) (12). Individual *CD34* ^+^ cells were enriched and picked up at days 4 (n=24), 6 (n=36), and 8 (n=48; Figure 2A). Because the complexity and percentage of *CD34* ^+^ cells increased during differentiation (15), more individual cells were analyzed at days 6 and 8. Because we used magnetic cell sorting to enrich *CD34* ^+^ cells, we could not catch cells with low or no expression of *CD34*, which were important for the hematopoietic differentiation study at the initial stage. All human cells (*CD34* positive or negative) derived from hPSCs can be analyzed by high-throughput single-cell RNA-sequencing in our further research, which will help us study the differentiation process before *CD34* ^+^ cells appear.

**Figure 3.**
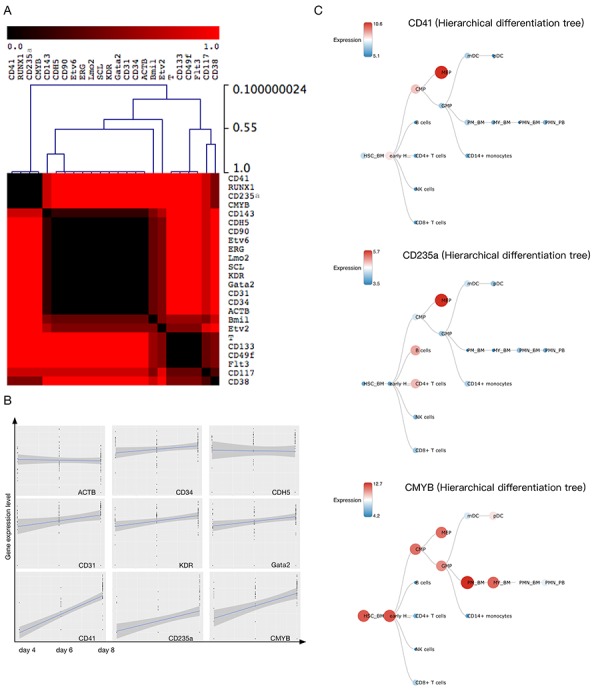
Gene-to-gene correlation shown by single-cell gene expression analysis. *A,* Heatmap of non-negative matrix factorization showing gene-to-gene correlation. The rank is 3. *B,* Gene expression (log2) trends from day 4 to day 8. *ACTB*, *CD34,* and *CDH5* were stable. *CD31*, *KDR,* and *Gata2* increased gradually. *CD41*, *CD235a*, and *CMYB* had a remarkable increase. *C,* Hierarchical differentiation tree of normal human hematopoiesis (HemaExplorer, BloodSpot) showing the relative expression of *CD41*, *CD235a*, and *CMYB* in different blood cells. Blue to white suggests low to moderate gene expression (log2) and white to red suggests moderate to high gene expression (log2). MEP: megakaryocyte-erythroid progenitor cell.

After single-cell specific target amplification, single-cell qPCR was performed to check the expression level of *CD34* and *ACTB.* We removed the samples without *CD34* expression, which corresponded to empty tubes or false positive cells. Then, we filtered samples based on the expression level of *ACTB* (Figure 2B). Lower and higher expression level of *ACTB* indicated RNA degradation and multicellular interference, respectively. After filtering, 91 samples (day 4, n=16; day 6, n=29; day 8, n=46) were qualified with stable expression trends of *ACTB* and *CD34* from days 4 to 8 (Figure 3B).

We used single-cell qPCR to measure the expression level of endothelial/hematoendothelial markers and hematopoietic TFs. Individual *CD34* ^+^ cells from days 4, 6, and 8 were marked with red, green, and blue respectively (Figure 2C and D). The heatmap of HCL showed that individual *CD34* ^+^ cells from different stages of differentiation were well clustered into 4 groups (A, B, C, and D; Figure 2D and E). The heatmap of NMF, which showed cell-to-cell correlation, also had a high score with factorization rank 4 (Figure 2C). Group A was composed of single-cell samples from day 4 (5/16=31.3%) and day 6 (6/29=20.7%; Figure 2E). The expression level of endothelial/hematoendothelial markers (*CD34*, *CDH5*, *KDR*, and *CD31*) and hematopoietic TFs (*Bmi1*, *Etv6*, *Lmo2*, *ERG*, *SCL*, *Gata2*, *CMYB*, etc.) was low in most of group A (Figure 2D). With few features of endothelial/hematoendothelial cells (24), these *CD34* ^+^ cells might be at the mesodermal-to-endothelial phase. Group B was composed of individual *CD34* ^+^ cells from days 4 (11/16=68.7%), 6 (16/29=55.2%), and 8 (7/46=15.2%; Figure 2E). Compared to group A, group B had a higher expression level of endothelial/hematoendothelial markers, which indicated the endothelial commitment (Figure 2D) (24); the expression level of hematopoietic TFs increased moderately. Compared to group B, both group C (day 6, 5/29=17.2%; day 8, 27/46=58.7%) and group D (day 6, 2/29=6.9%; day 8, 12/46=26.1%; Figure 2E) had higher expression levels of hematopoietic TFs (Figure 2D). Group D did not express *CDH5* and may indicate the commitment of hematopoietic progenitors from hematoendothelial cells.

The gene heatmap of NMF with factorization rank 3 shows the gene-to-gene correlation (Figure 3A). *CD41* (a marker of megakaryocytes), *CD235a*, and *CMYB* were clustered together because of their similar expression dynamics (Figure 3A). They were highly expressed in both groups C and D with similar cell distribution (Figure 2D); their expression trends from days 4 to 8 were similar (Figure 3B). This relationship could also be found in previous reports. Double positive of *CD41* and *CD235a* was used to define erythro-megakaryocytic progenitors (E/Mk-HP) as reported previously (16). BloodSpot database also showed that *CD41*, *CD235a*, and *CMYB* were mainly located in E/Mk-HP of healthy samples (25) (Figure 3C). Both groups C and D had individual cells with high expression levels of *CD41* and *CD235a*, but the expression distribution of *CD31* and *CDH5* was different in groups C and D. It is possible that *CD41* ^+^ *CD235a* ^+^ *CD31* ^+^ *CDH5* ^+^ (C group) cells down regulated the expression of *CD31* and *CDH5* to become *CD41* ^+^ *CD235a* ^+^ *CD31* ^-^ *CDH5* ^-^ (D group) cells in the process of differentiation and maturation.

We also found that some hematopoietic TFs were gradually up-regulated during the differentiation (Figure 2D). OP9 coculture system was enough to up-regulate these TFs at day 8. We wanted to explore the possibility that overexpression of these hematopoietic TFs at early stage of differentiation could improve the efficiency of hematopoietic differentiation in this coculture system.

### Induced expression of hematopoietic transcription factors enhanced hematopoietic differentiation

**Figure 4.**
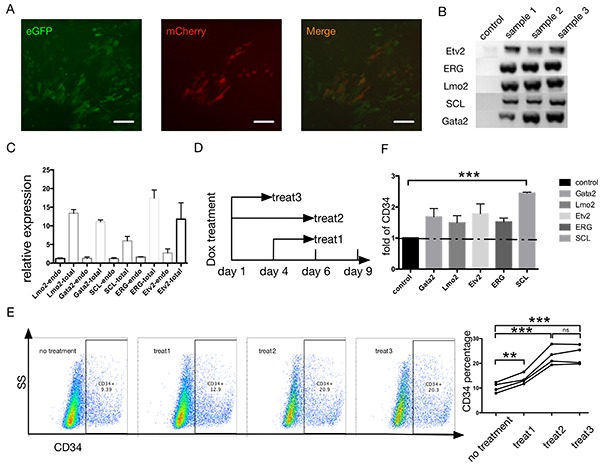
Induced expression of hematopoietic transcription factors enhanced hematopoietic differentiation. *A,* H1 cells transduced by Lv-ef1α-eGFP-tre-mCherry were green. Merged cells were transduced by both Lv-ef1α-rtTA-IRES-puro and Lv-ef1α-eGFP-tre-mCherry. The expression of mCherry (red) was induced after 48 h of Dox treatment. Scale bar=100 μm. *B,* Genomic PCR was used to confirm the exogenous genes integration. Control was H1 cells transduced by mCherry (Lv-ef1α-eGFP-tre-mCherry). *C,* Samples were treated with 1 μg/mL doxycycline (Dox) for 48 h. qPCR was used to confirm the relative expression of endogenous and exogenous genes. endo: endogenous expression; total: endogenous plus exogenous expression. Data are reported as means±SE (n≥3). *D,* Strategies of Dox treatment. Treat1: Dox was added at days 4–6; treat2: Dox was added at days 1–6; treat3: Dox was added at days 1–4. *E,* Percentage of *CD34* ^+^ cells in different treatment groups (no treatment, treat1, treat2, and treat3) at day 9 (n≥4). The difference was compared using paired *t*-tests. *F,* The effect of individual transcription factors on the proportion of *CD34* ^+^ cells. Data are reported as fold of increase for n≥3. **P*<*0.01, ***P<0.001 (*t*-test).

The TFs were overexpressed using Tet-On inducible expression system as shown in our previous papers (22,23). This approach possessed the advantage that hPSCs could retain their pluripotency and self-renewal ability after lentivirus transduction; exogenous genes did not express until Dox was added. After 48 h of Dox treatment, we induced the expression of mCherry in H1 transduced by mCherry lentivirus (Figure 4A). We assembled a list of key TFs through analysis of our single-cell gene expression data (Figure 2D) and literature review (26–28). The pilot experiments showed serious death of hPSCs after excessive transduction of 18 exogenous genes. Results suggested that hPSCs were more sensitive than fibroblasts to the stress of lentiviral transduction (29). Thus, we narrowed the TFs list to five (*Gata2*, *Lmo2*, *Etv2*, *ERG*, *SCL*), which were used in other hematopoietic differentiation strategies (26,27,30 [Bibr B31] [Bibr B32] [Bibr B33] [Bibr B34]).

After lentiviral transduction, H1 cells were collected for genomic PCR to confirm the integration of exogenous genes (Figure 4B), which were induced by Dox treatment (Figure 4C). We found that samples exposed to Dox had fewer cells than control groups, suggesting that overexpression of exogenous genes induced cell death or cell differentiation with proliferation inhibition. To reduce the adverse impact of overexpression, we optimized the treatment time of Dox. We added Dox at different stages of differentiation, including treat1, treat2, and treat3 (Figure 4D). Dox treatment increased the percentage of *CD34* ^+^ cells significantly (Figure 4E). Both treat2 and treat3 had higher proportion of *CD34* ^+^ cells than no treatment and treat1. We got 0.8-1×10^5^ *CD34* ^+^ cells from no treat group, and 2-3×10^5^ *CD34* ^+^ cells from treat2 and treat3, demonstrating that Dox treatment from days 1 to 4 (treat2 and treat3) was crucial for differentiation of *CD34* ^+^ cells. Treat2 with additional Dox treatment did not generate more *CD34* ^+^ cells than treat3 (Figure 4E). Our single-cell gene expression analysis showed that OP9 system could up-regulate most of these TFs after day 4 (Figure 2D). Then, we inferred that overexpression of these exogenous genes mainly promoted the differentiation at the initial stage (before day 4). Of note, continuous overexpression could not improve hematopoietic differentiation anymore and may disrupt the expression of endogenous genes and differentiation signals provided by OP9. Based on these results and analysis, we added Dox as treat3 in the following experiments.

We tried to induce hematopoietic differentiation from monolayer hPSCs without OP9, resulting in more *CD34* ^+^ cells with only 4 days differentiation (Supplementary Figure S1). H1 cells without TFs overexpression had few *CD34* ^+^ cells (∼1%), and H1 with TFs overexpression had 20∼30% *CD34* ^+^ cells, suggesting that these transcription factors were very important during hematopoietic differentiation as previous papers have reported (26,27,30).

Mixed TFs cover up the effect of individual TFs. We found that individual TFs can increase the percentage of CD34^+^ cells, especially *SCL* (Figure 4F). Many other hematopoietic TFs have been reported in hematopoietic differentiation studies (28,). Therefore, we should screen more TFs combinations (single and mixed) and overexpression strategies in further research. By combining single-cell gene expression analysis, we can study the effect of single or mixed TFs in the process of hematopoietic differentiation, and optimize our hematopoietic differentiation system.

In our study, exogenous TFs integrated into the genome of hPSCs by lentiviral transduction. In a previous study, an exogenous gene (*Lhx2*) was overexpressed in OP9 (OP9-Lhx2) (35) to enhance hematopoietic differentiation of hPSCs. This strategy avoids the tumorigenic risk of blood cells derived from hPSCs with lentivirus integration. It is generally agreed that tumorigenic risk needs to be taken seriously in the differentiation of tissue cells for replacement therapy, such as long-lived HSCs, neural cells, and muscle cells. Mature blood cells have a relatively short life span. As most clinical blood products (36), mature red blood cells and platelets do not have nuclei. Therefore, the risk of tumorigenesis caused by lentivirus integration is not so serious in the differentiation of hematopoietic progenitors for the generation of mature blood cells *in vitro*.

### Expressional and functional analysis of CD34+ cells with transcription factors overexpression

Hematopoietic TFs played a decisive role in hematopoietic differentiation. Different combination of TFs induced differentiation of different hematopoietic progenitors (26). Though exogenous TFs were induced only at early stage of differentiation, we did not know whether the fate of *CD34* ^+^ cells was disturbed by these TFs overexpression. Thus, we needed to check the differentiation potential of these *CD34* ^+^ cells.

**Figure 5.**
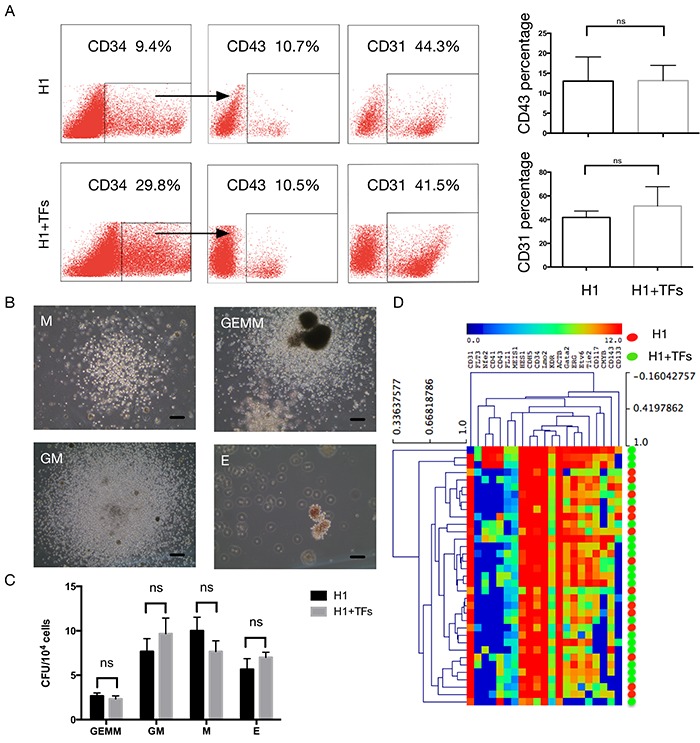
Expression and functional analysis of *CD34* ^+^ cells after transcription factors overexpression. *A,* Flow cytometry showing the percentage of *CD43* ^+^ cells and *CD31* ^+^ cells in *CD34* ^+^ cells after transcription factors overexpression. H1: cells without lentiviral transduction; H1+TFs: H1 transduced by lentivirus; n≥3. ns: P>0.05 (*t*-test). *B* and *C,* Morphology of different colony-forming unit (CFU) types after transcription factors overexpression, including M, GM, GEMM, and E. Scale bar=100 μm; n≥3. ns: P>0.05 (*t*-test). *D,* Heatmap of hierarchical clustering showing the expression characteristics of individual *CD34* ^+^ from day 8. Red and green colors of each row correspond to individual *CD34* ^+^ cells from H1 and H1+TFs group respectively. Each column corresponds to a specific gene. Color scale was set from 0 to 12. Blue to green suggests low to moderate gene expression (log2) and green to red suggests moderate to high gene expression (log2).

Compared to *CD34*, *CD43* was a better marker to define hematopoietic progenitors in hPSCs/OP9 coculture system (15,16). Before *CD45* expression, *CD43* was expressed in all types of emerging progenitors. *CD43* was used to separate hematopoietic progenitors from endothelial cells (*CD34* ^+^ *CD43* ^-^ *CD31* ^+^ *KDR* ^+^) and mesenchymal cells (*CD34* ^+^ *CD43* ^-^ *CD31* ^-^ *KDR* ^-^) (15). After Dox treatment, there was no significant difference between overexpression group and control group in the percentage of *CD43* ^+^ cells and *CD31* ^+^ cells at day 8 (Figure 5A). CFU assays also showed normal differentiation potency of *CD34* ^+^ cells with TFs overexpression (Figure 5B and C). Single-cell gene expression analysis also showed similar gene expression characteristics of *CD34* ^+^ cells (H1: n=14. H1+TFs: n=21) at day 8 (Figure 5D), such as CD43, CD31, *CD117*, and *CDH5*. After overexpression, we generated more *CD34* ^+^ cells with normal differentiation potency for further myeloid cells and erythroid cells differentiation. Transient overexpression of specific hematopoietic TFs at early stage of differentiation did not disrupt the differentiation signals provided by OP9.

Previous reports showed that HSCs had lineage biases before lineage differentiation (37,38). Overexpression of TFs may generate hematopoietic progenitors with lineage biases, which prefer to produce one or two types of mature blood cells. Unipotent hematopoietic progenitors are important for the production of mature blood cells for clinical use. Through single-cell analysis of defined mature blood cells derived from hPSCs with a random integration of exogenous genes, we may reveal the relationships between specific exogenous genes integration and lineage biases of hematopoietic progenitors.

In conclusion, single-cell gene expression analysis revealed the dynamic gene expression of individual *CD34* ^+^ cells from different stages of differentiation in hPSCs/OP9 coculture system. Results provided guidance for optimization of hematopoietic differentiation. High-throughput single-cell analysis, including single-cell qPCR and single-cell RNA-sequencing, can provide better gene expression profiles of OP9 and hPSCs for optimization of hematopoietic differentiation in hPSCs/OP9 coculture system.

## Supplementary Material

Click here to view [pdf]

## References

[1] Thomson JA, Itskovitz-Eldor J, Shapiro SS, Waknitz MA, Swiergiel JJ, Marshall VS (1998). Embryonic stem cell lines derived from human blastocysts. Science.

[B02] Yu JY, Vodyanik MA, Smuga-Otto K, Antosiewicz-Bourget J, Frane JL, Tian S (2007). Induced pluripotent stem cell lines derived from human somatic cells. Science.

[3] Takahashi K, Tanabe K, Ohnuki M, Narita M, Ichisaka T, Tomoda K (2007). Induction of pluripotent stem cells from adult human fibroblasts by defined factors. Cell.

[4] Dye BR, Hill DR, Ferguson MA, Tsai YH, Nagy MS, Dyal R (2015). *In vitro* generation of human pluripotent stem cell derived lung organoids. eLife.

[5] Hayashi R, Ishikawa Y, Katori R, Sasamoto Y, Taniwaki Y, Takayanagi H (2017). Coordinated generation of multiple ocular-like cell lineages and fabrication of functional corneal epithelial cell sheets from human iPS cells. Nat Protoc.

[6] Spence JR, Mayhew CN, Rankin SA, Kuhar MF, Vallance JE, Tolle K (2011). Directed differentiation of human pluripotent stem cells into intestinal tissue *in vitro*. Nature.

[7] Zhong X, Gutierrez C, Xue T, Hampton C, Vergara MN, Cao LH (2014). Generation of three-dimensional retinal tissue with functional photoreceptors from human iPSCs. Nat Commun.

[8] Takebe T, Zhang RR, Koike H, Kimura M, Yoshizawa E, Enomura M (2014). Generation of a vascularized and functional human liver from an iPSC-derived organ bud transplant. Nat Protoc.

[9] Choi KD, Vodyanik M, Slukvin II (2011). Hematopoietic differentiation and production of mature myeloid cells from human pluripotent stem cells. Nat Protoc.

[10] Slukvin II (2013). Hematopoietic specification from human pluripotent stem cells: current advances and challenges toward de novo generation of hematopoietic stem cells. Blood.

[11] Wahlster L, Daley GQ (2016). Progress towards generation of human haematopoietic stem cells. Nat Cell Biol.

[12] Vodyanik MA, Bork JA, Thomson JA, Slukvin II (2005). Human embryonic stem cell-derived CD34+ cells: efficient production in the coculture with OP9 stromal cells and analysis of lymphohematopoietic potential. Blood.

[13] Dias J, Gumenyuk M, Kang H, Vodyanik M, Yu J, Thomson JA (2011). Generation of red blood cells from human induced pluripotent stem cells. Stem Cells Dev.

[14] Figueiredo LM, Costa EB, Orellana MD, Picanco-Castro V, Covas DT (2015). OP9 stromal cells proteins involved in hematoendothelial differentiation from human embryonic stem cells. Cell Reprogram.

[15] Vodyanik MA, Thomson JA, Slukvin II (2006). Leukosialin (CD43) defines hematopoietic progenitors in human embryonic stem cell differentiation cultures. Blood.

[16] Salvagiotto G, Zhao Y, Vodyanik M, Ruotti V, Stewart R, Marra M (2008). Molecular profiling reveals similarities and differences between primitive subsets of hematopoietic cells generated in vitro from human embryonic stem cells and in vivo during embryogenesis. Exp Hematol.

[17] Yan L, Yang M, Guo H, Yang L, Wu J, Li R (2013). Single-cell RNA-Seq profiling of human preimplantation embryos and embryonic stem cells. Nat Struct Mol Biol.

[18] Guo G, Luc S, Marco E, Lin TW, Peng C, Kerenyi MA (2013). Mapping cellular hierarchy by single-cell analysis of the cell surface repertoire. Cell Stem Cell.

[19] Moore FE, Garcia EG, Lobbardi R, Jain E, Tang Q, Moore JC (2016). Single-cell transcriptional analysis of normal, aberrant, and malignant hematopoiesis in zebrafish. J Exp Med.

[20] Lu P, Chen J, He L, Ren J, Chen H, Rao L (2013). Generating hypoimmunogenic human embryonic stem cells by the disruption of beta 2-microglobulin. Stem Cell Rev.

[21] Cui D, Wang J, Zeng Y, Rao L, Chen H, Li W (2016). Generating hESCs with reduced immunogenicity by disrupting TAP1 or TAPBP. Biosci Biotechnol Biochem.

[22] Rao L, Tang W, Wei Y, Bao L, Chen J, Chen H (2012). Highly efficient derivation of skeletal myotubes from human embryonic stem cells. Stem Cell Rev.

[23] Wu Z, Chen J, Ren J, Bao L, Liao J, Cui C (2009). Generation of pig induced pluripotent stem cells with a drug-inducible system. J Mol Cell Biol.

[24] Zambidis ET, Peault B, Park TS, Bunz F, Civin CI (2005). Hematopoietic differentiation of human embryonic stem cells progresses through sequential hematoendothelial, primitive, and definitive stages resembling human yolk sac development. Blood.

[25] Bagger FO, Sasivarevic D, Sohi SH, Laursen LG, Pundhir S, Sonderby CK (2016). BloodSpot: a database of gene expression profiles and transcriptional programs for healthy and malignant haematopoiesis. Nucleic Acids Res.

[26] Elcheva I, Brok-Volchanskaya V, Kumar A, Liu P, Lee JH, Tong L (2014). Direct induction of haematoendothelial programs in human pluripotent stem cells by transcriptional regulators. Nat Commun.

[27] Doulatov S, Vo LT, Chou SS, Kim PG, Arora N, Li H (2013). Induction of multipotential hematopoietic progenitors from human pluripotent stem cells via respecification of lineage-restricted precursors. Cell Stem Cell.

[28] Sandler VM, Lis R, Liu Y, Kedem A, James D, Elemento O (2014). Reprogramming human endothelial cells to haematopoietic cells requires vascular induction. Nature.

[29] Takahashi K, Yamanaka S (2006). Induction of pluripotent stem cells from mouse embryonic and adult fibroblast cultures by defined factors. Cell.

[30] Batta K, Florkowska M, Kouskoff V, Lacaud G (2014). Direct reprogramming of murine fibroblasts to hematopoietic progenitor cells. Cell Rep.

[B31] Szabo E, Rampalli S, Risueno RM, Schnerch A, Mitchell R, Fiebig-Comyn A (2010). Direct conversion of human fibroblasts to multilineage blood progenitors. Nature.

[B32] Pereira CF, Chang B, Qiu J, Niu X, Papatsenko D, Hendry CE (2013). Induction of a hemogenic program in mouse fibroblasts. Cell Stem Cell.

[B33] Harris DM, Hazan-Haley I, Coombes K, Bueso-Ramos C, Liu J, Liu Z (2011). Transformation of human mesenchymal cells and skin fibroblasts into hematopoietic cells. PLoS One.

[B34] Riddell J, Gazit R, Garrison BS, Guo G, Saadatpour A, Mandal PK (2014). Reprogramming committed murine blood cells to induced hematopoietic stem cells with defined factors. Cell.

[35] Chen X, Zhao Q, Li C, Geng Y, Huang K, Zhang J (2015). OP9-Lhx2 stromal cells facilitate derivation of hematopoietic progenitors both *in vitro* and *in vivo*. Stem Cell Res.

[36] Slukvin II (2010). Generation of mature blood cells from pluripotent stem cells. Haematologica.

[37] Velten L, Haas SF, Raffel S, Blaszkiewicz S, Islam S, Hennig BP (2017). Human haematopoietic stem cell lineage commitment is a continuous process. Nat Cell Biol.

[38] Notta F, Zandi S, Takayama N, Dobson S, Gan OI, Wilson G (2016). Distinct routes of lineage development reshape the human blood hierarchy across ontogeny. Science.

